# Clinical features of concurrent systemic lupus erythematosus and renal biopsy-proven antineutrophil cytoplasmic antibody-associated vasculitis: a retrospective analysis

**DOI:** 10.3389/fmed.2026.1842903

**Published:** 2026-09-09

**Authors:** Li-Yuan Xie, Xian-Ying Qiu, Hao-Miao Zhang, Chen-Xi Cai, Di Zhang, Peng-Cheng Xu, Jun-Ya Jia

**Affiliations:** 1 Department of Nephrology, Tianjin Medical University General Hospital, Tianjin, China; 2 Department of Nephrology, The People’s Hospital of Guangxi Zhuang Autonomous Region, Guangxi Clinical Research Center for Chronic Kidney Disease, Nanning, China

**Keywords:** antineutrophil cytoplasmic antibodies, antineutrophil cytoplasmic antibody-associated glomerulonephritis, antineutrophil cytoplasmic antibody-associated vasculitis (AAV), SLE/AAV overlap syndrome, systemic lupus erythematosus (SLE)

## Abstract

**Background:**

Antineutrophil cytoplasmic antibody (ANCA) positivity is not uncommon in patients with systemic lupus erythematosus (SLE), however, the diagnosis of concurrent SLE and ANCA-associated vasculitis (AAV) is challenging because SLE tends to mask the clinical manifestations of AAV. Since the kidney is the most frequently affected organ in AAV, we consider that SLE patients with concurrent renal biopsy-proven AAGN warrant the greatest clinical attention.

**Methods:**

We enrolled two patients with newly diagnosed SLE and renal biopsy-proven AAV glomerulonephritis (AAGN). After a literature search, another 57 similar patients were identified. Then the clinical characteristics of these patients were analyzed.

**Results:**

Including the two newly reported patients, a total of 51 female and 8 male patients were included. Compared with patients with AAGN alone, patients with combined lupus nephritis (LN) and AAGN had higher SLEDAI scores [14.00 (10.00–18.00) vs. 5.00 (2.50–12.50), *P* = 0.028], and the 24-h urinary protein level tended to be higher in the LN + AAGN group [3.10 (1.70, 6.69) g/d vs. 1.66 (0.88, 2.15) g/d, *p* = 0.066]. Patients with elevated C-reactive protein (CRP) levels had lower SLEDAI scores (*P* = 0.021), higher leukocyte counts (*P* = 0.005). Patients with elevated leukocyte counts had the lowest proteinuria levels (*P* = 0.029) but the highest incidence of pulmonary hemorrhage (*P* = 0.016) compared to those with normal or decreased counts. Low complement 4 and high proteinuria levels were associated with high SLEDAI scores (*P* = 0.041 and *P* = 0.027, respectively). Patients who progressed to end-stage renal disease had a higher mortality than those who achieved renal remission (*P* < 0.001). Renal prognosis did not differ between LN + AAGN and AAGN alone groups (23.1% vs. 25.0%, *p* = 1.000). The mortality rate was 23.1% in the LN + AAGN group and 5.0% in the AAGN alone group (*P* = 0.166).

**Conclusion:**

For ANCA-positive SLE patients, renal injury may be caused by LN, LN combined with AAGN, or even isolated AAGN. So renal biopsy plays a crucial role in guiding the accurate choice of therapeutic strategy. Further studies are required owing to the heterogeneity of case sources.

## 1 Introduction

Antineutrophil cytoplasmic antibodies (ANCA) are widely recognized as markers for antineutrophil cytoplasmic antibody-associated vasculitis (AAV). However, a high prevalence of ANCA positivity is also observed in other autoimmune diseases, such as rheumatoid arthritis, inflammatory bowel disease, and systemic lupus erythematosus (SLE) (1). Determining the significance of ANCA in non-AAV diseases is challenging. Previous reports indicate that the rate of ANCA positivity in SLE ranges from 13% to 37%. Some research suggests that ANCA-positive SLE may present with more severe clinical manifestations than ANCA-negative SLE (2–6). Because the presence of multiple autoantibodies is a hallmark of SLE, it can be challenging to determine whether ANCA-positive SLE indicates a coexistence of SLE with AAV or simply reflects a state of highly active SLE. Some studies have reported cases of “SLE/AAV overlap syndrome”, but most diagnoses of SLE and AAV were made successively rather than simultaneously (7, 8). Theoretically speaking, patients with SLE/AAV occurring successively and those with SLE/AAV occurring simultaneously have significant differences in clinical features. Another concern is that the majority of patients with concurrent SLE/AAV reported in the existing literature lack pathological evidence, which undermines the accuracy of AAV diagnosis. In fact, the diagnosis of AAV in the vast majority of such patients remains questionable.

In summary, the concurrent occurrence of SLE and renal biopsy-confirmed AAV represents an extremely rare clinical entity. A review of our center's longitudinal data identified only two such cases, precluding the feasibility of a single-center cohort analysis. Accordingly, we incorporated previously reported cases from the literature for systematic analysis (7–48). Despite the small total sample size of 59 patients and the heterogeneity across multiple influencing factors among these patients, several meaningful conclusions were still derived.

## 2 Materials and methods

### 2.1 Data sources and search strategy

We retrieved cases of SLE with biopsy-proven AAGN through the database of Tianjin Medical University General Hospital. Medical charts were reviewed independently by two experts to validate the diagnosis of overlap syndrome. At last, two patients were included. This study was conducted according to the principles expressed in the Declaration of Helsinki. Both patients had provided written informed consent before the data collection.

We also searched the literature in PubMed, Web of Science, Embase, China National Knowledge Infrastructure, and WANFANG DATA to identify relevant literature published between January 1990 and July 2026. Two investigators (Xie LY and Qiu XY) independently conducted the search using a predefined set of keywords to ensure comprehensive coverage of the literature. An example search string was: (((Systemic Lupus Erythematosus OR Lupus) AND (Anti-Neutrophil Cytoplasmic Antibody-Associated Vasculitis OR ANCA-associated vasculitis OR Antineutrophil Cytoplasmic Antibody OR ANCA OR granulomatosis with polyangiitis OR Wegener's granulomatosis OR microscopic polyangiitis OR eosinophilic granulomatosis with polyangiitis OR Churg-Strauss syndrome OR pauci-immune glomerulonephritis OR crescentic glomerulonephritis)) OR (SLE/AAV overlap syndrome)) AND [(“1990/01/01” [Date—Publication]: “2026/07/01” [Date—Publication])]. The initial search yielded 9720 articles; after deduplication, 143 remained for further analysis. Subsequently, the same two authors (Xie LY and Qiu XY) carefully reviewed the titles, abstracts, and full texts of the remaining papers, reaching consensus on inclusion assessment. The comprehensive literature review ultimately identified 50 articles, documenting 57 cases of SLE with biopsy-proven AAGN.

**Figure 1:**
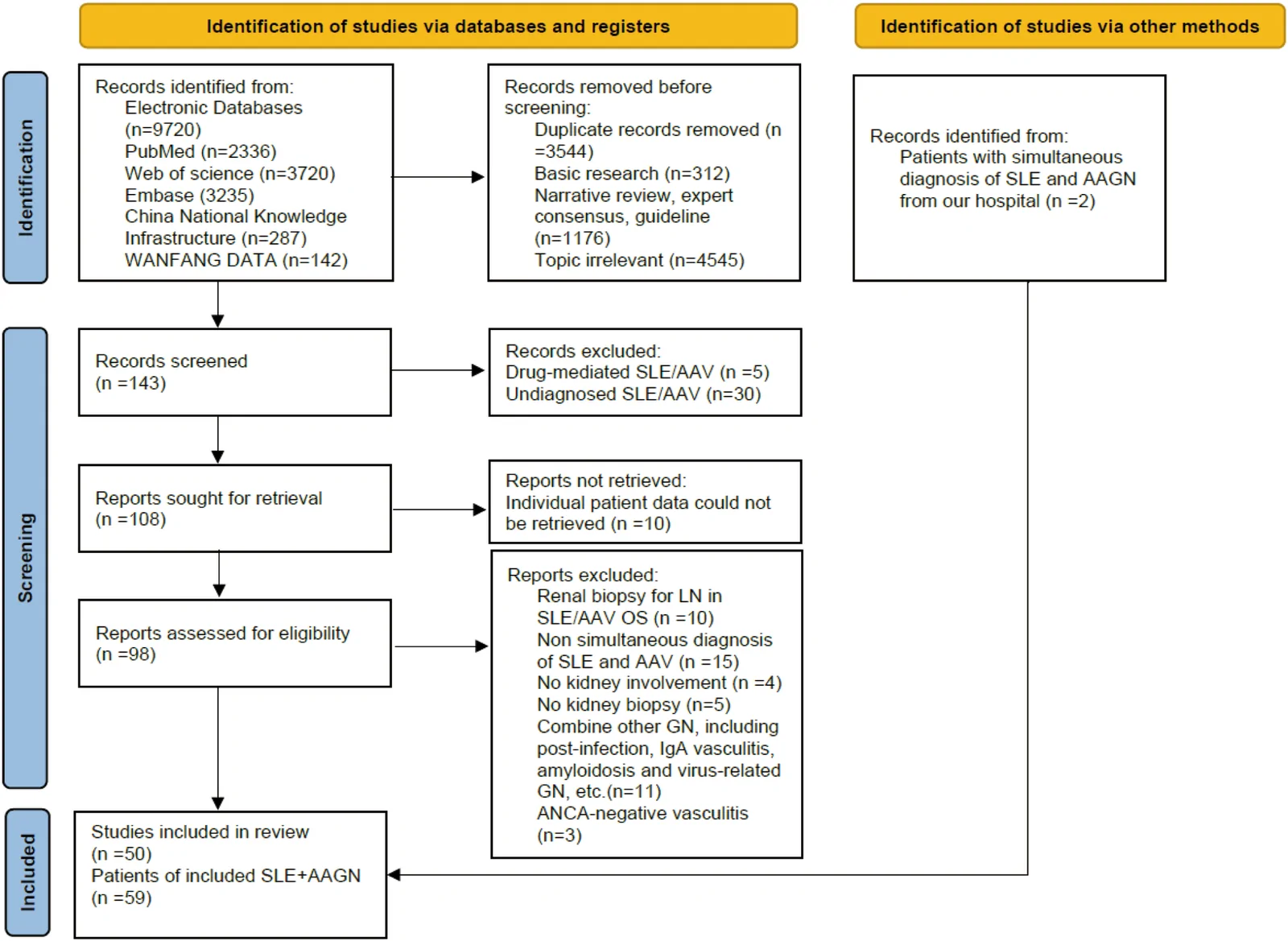
PRISMA flow diagram. ANCA, anti-neutrophil cytoplasmic antibody; AAV, ANCA-associated vasculitis; AAGN, ANCA-associated glomerulonephritis; SLE, systemic lupus erythematosus.

Combining the hospital records and the literature review, a total of 59 cases of SLE with biopsy-proven AAGN were obtained, as shown in Figure 1.

### 2.2 Study selection and eligibility criteria

Two authors (Xie LY and Qiu XY) independently screened and selected studies/patients for inclusion. We simultaneously included published studies (including case reports, reviews, original articles and other literature types) with extractable raw clinical data of patients, as well as patients of SLE with biopsy-proven AAGN screened from the database of Tianjin Medical University General Hospital. We included studies/patients that met the following criteria: (1) publications were included if raw patient data (such as age, sex, clinical manifestations, laboratory tests and renal biopsy results) were extractable; (2) patients were adults or children (no age restriction); (3) concurrent diagnosis of AAV and SLE, meeting the sets of diagnostic/classification criteria. For SLE: the 1997 American College of Rheumatology (ACR) classification criteria (Meeting 4 or more items) or the 2019 classification criteria jointly developed by the European League Against Rheumatism (EULAR) and ACR (Positive for antinuclear antibody with a total score >= 10) (49, 50); For AAV: the 2012 revised Chapel Hill Consensus Conference definition criteria for AAV or the classification criteria jointly published in 2022 by the ACR and the European Alliance of Associations for Rheumatology (EULAR) (51–54); (4) presence of ANCA-associated glomerulonephritis (AAGN) confirmed by renal biopsy, with or without lupus nephritis (LN). According to the definition in previous literature, patients who meet the internationally recognized classification criteria for both SLE and AAV, either simultaneously or sequentially, are defined as having SLE/AAV overlap syndrome (7, 9, 16, 22, 48). Exclusion criteria: (1) Narrative reviews and basic research, such as animal studies, cell experiments and pharmacokinetic research; (2) Studies with topics unrelated to SLE/AAV overlap syndrome; (3) Studies lacking raw clinical data of patient. (4) Drug-induced SLE/AAV; (5) SLE and AAV occur sequentially, not concurrently; (6) No kidney involvement; No renal biopsy performed; (7) combine other glomerulonephritis, including post-infectious glomerulonephritis, IgA vasculitis, amyloidosis, and virus-related glomerulonephritis, etc.; (8) ANCA-negative vasculitis.

### 2.3 Data extraction

Data extracted from eligible studies were recorded in Microsoft Excel spreadsheets. Demographic, clinical data and clinical manifestations were recorded, such as age, sex, race, and medical history of autoimmune diseases, general symptoms (fever, weight loss), cutaneous lesions, ear–nose–throat involvement, ophthalmologic, articular, neurologic, cardiac, pulmonary, and vascular involvement. Disease activity was assessed using the SLE Disease Activity Index (SLEDAI) and the Birmingham Vasculitis Activity Score (BVAS). Laboratory data were collected as follows: white blood cell (WBC) count, platelet count, hemoglobin, C-reactive protein (CRP), erythrocyte sedimentation rate (ESR), serum creatinine, 24-h proteinuria and urinary sediment, antinuclear antibodies (ANA), anti-double-stranded DNA (dsDNA), ANCA serotype and titer, antiphospholipid antibodies, and complement 3 (C3) and complement 4 (C4) levels. The estimated glomerular filtration rate (eGFR) was calculated using the Chronic Kidney Disease Epidemiology Partnership study equation. The therapeutic regimen, pathological classification, renal biopsy pathological features and other relevant indicators were also documented.

### 2.4 Diagnosis and classification of AAGN in renal biopsies

Kidney biopsies were analyzed with light microscopy, electron microscopy, and immunofluorescence (IF). The percentages of sclerotic and crescentic glomeruli, as well as the presence of endocapillary proliferation, were detailed for each biopsy. Subendothelial, mesangial, and subepithelial immune complex deposits (Immunoglobulin G, Immunoglobulin M, IgA, C3, and complement 1q) were semi-quantitatively assessed, ranging from 0 to 4+.

When there was no pathological evidence of co-existing LN, AAGN was diagnosed according to the following criteria: under light microscopy, glomerular crescents were observed, which could be accompanied by fibrinoid necrosis or granulomas. Immunofluorescence examination indicated that immunoglobulins and complements were negative or only weakly positive, and electron microscopy showed no evident electron-dense deposits.

When LN coexisted with AAGN, the diagnosis of AAGN relied on the following features: (1) the extent of crescents and fibrinoid necrosis was significantly more severe than the degree of mesangial and endothelial proliferation in the glomerulus; (2) granuloma formation, a typical feature of AAGN, was present; (3) fibrinoid necrosis was present in extraglomerular vessels in addition to the glomerulus.

AAGN classification followed the Berden 2010 criteria: (1) focal: ≥50% normal glomeruli; (2) crescentic: ≥50% glomeruli with cellular crescents; (3) mixed: <50% normal, <50% crescentic, <50% globally sclerotic glomeruli; (4) sclerotic: ≥50% globally sclerotic glomeruli.

### 2.5 Identification and classification of the co-existing LN

The coexistence of LN is diagnosed based on the following pathological features: (1) immunofluorescence staining exhibits a “full house” pattern, characterized by strong positive expression of Immunoglobulin G(IgG), Immunoglobulin M(IgM), IgA, C3, complement 1q(C1q), and fibrin; (2) under light microscopy, characteristic LN features such as endocapillary proliferation (mesangial and endothelial cell proliferation), infiltration of polymorphonuclear neutrophils, and the formation of wire loops or crescents are observed; (3) electron microscopy reveals electron-dense deposits in various parts of the glomerulus (mesangial area, subendothelial and subepithelial regions), and occasionally in the renal tubules or interstitium.

LN pathological classification follows the 2003 International Society of Nephrology/Renal Pathology Society (ISN/RPS) criteria, which categorizes LN into the following classes: Class I, minimal mesangial LN; Class II, mesangial proliferative LN; Class III, focal LN; Class IV, diffuse LN; Class V, membranous LN; and Class VI, advanced sclerosing LN.

When AAGN coexists with LN, the formation of crescents and fibrinoid necrosis is often increased. On the other hand, mesangial proliferation and endothelial cell proliferation are considered key parameters for classifying LN. Therefore, glomeruli showing only crescents or fibrinoid necrosis, without significant mesangial or endothelial proliferation, are attributed to AAGN rather than LN.

### 2.6 Statistics

Statistical analyses were performed using SPSS version 26.0 (IBM Corp, Armonk, NY, USA) and GraphPad Prism 8 (version 8.0.2; GraphPad Software, La Jolla, CA, USA). Continuous variables were expressed as mean ± standard deviation or median (interquartile range, IQR), depending on the data distribution. Categorical variables were described using frequency and percentage. Normality was assessed with the single-sample Shapiro–Wilk test. For group comparisons, the *t*-test or Mann–Whitney *U* test was applied to continuous variables, depending on data distribution, and the chi-square test or Fisher's exact test was used for categorical variables. Spearman's rank correlation coefficient was used to assess correlations between the BVAS and laboratory data. Survival was estimated using the Kaplan–Meier method, and differences were evaluated using a stratified log-rank test. Statistical significance was defined as *P* < 0.05. The receiver operating characteristic (ROC) curve and the area under the curve (AUC) were used to assess the model's discrimination ability.

## 3 Results

### 3.1 Study and patient characteristics

Between 2021 and 2023, two patients with SLE combined with AAGN were diagnosed in our center. One patient presented with both LN and AAGN (LN + AAGN), while the other exhibited AAGN without LN. Detailed information about the two patients is provided in Table 1. Following a literature search, 57 additional patients were enrolled. In subsequent analyses, data from all 59 patients were examined together (Figure 1). A total of 51 female and 8 male patients were included. The median age was 46.8 years (range, 12–80). The ethnic distribution was 38.98% Asian (*n* = 23), 50.85% Caucasian (*n* = 30), and 11.17% African (*n* = 6). Eight cases (6 Asian and 2 Caucasian) had a history of other autoimmune diseases, including Sjögren's syndrome, idiopathic thrombocytopenic purpura (ITP), catastrophic antiphospholipid syndrome (CAPS), autoimmune hepatitis (AIH), autoimmune thyroid disorders and psoriasis. The proportion of Asian patients with a history of autoimmune diseases (26.10%) tended to be higher than that of Caucasian (6.70%) and African patients (0.00%) (*P* = 0.073). All patients exhibited renal involvement, with 18 meeting the diagnostic criteria for nephrotic syndrome and 14 progressing to end-stage renal disease (ESRD). The observed extra-renal manifestations primarily included pulmonary (*n* = 33), muscle/articular (*n* = 26), mucocutaneous (*n* = 14), neurologic (*n* = 7), and ear–nose–throat (*n* = 4) involvement. Intra-alveolar hemorrhage was reported in 15 cases. ANA positivity was observed in almost all patients, with a median titer of 1:379 (range, 40–5120). All patients were also ANCA-positive, with predominantly myeloperoxidase (MPO)-ANCA positivity (*n* = 54), one case of proteinase 3 (PR3)-ANCA positivity, and four cases of dual positivity for both MPO-ANCA and PR3-ANCA.

**Table 1:** Characteristics of two patients diagnosed with LN + AAGN at our center.

	Case1	Case2
	55y, F	68y, M
Clinical manifestations	Fever, weight loss, leukopenia, anemia, nephrotic syndrome	Fever, weight loss, arthralgia, thrombocytopenia, interstitial pulmonary fibrosis
History of autoimmune disease	No	No
Biological tests	—	—
WBC (×10^9^/L)	2.76	4.86
Platelets (×10^9^/L)	164	67
Hemoglobin (g/L)	70	137
CRP (mg/dL)	0.18	1.43
C3/C4(mg/dL)	57.7/11.8	71.1/21.9
Creatinine (µmol/L)	285	359
Proteinuria (g/24 h)	9.5	0.875
ANA titer	1:160	1:320
Anti-dsDNA	+	+
ANCA	MPO-ANCA (189.26 IU/mL)	MPO-ANCA (260 IU/mL)
Renal biopsy	Focal proliferative and necrotizing GN with numerous crescents; Mesangial IgA±, IgG+, IgM+, C3+ and C1q+ deposits.	Focal proliferative and necrotizing GN with numerous crescents; Only C3(+) depositing along the loops of the glomerular capillaries, IgA, IgG, IgM, C4 and C1q (−).
Therapy	Methylprednisolone + tacrolimus	Glucocorticoid + cyclophosphamide
Follow-up	ESRD after 18 months	Renal remission

WBC, white blood cells; CRP, C-reactive protein; C3, complement 3; C4, complement 4; ANA, antinuclear antibody; dsDNA, double-stranded DNA; ANCA, antineutrophil cytoplasmic antibodies; MPO, myeloperoxidase.

### 3.2 Comparison of clinical and laboratory features between patients with LN + AAGN and those with AAGN alone

Based on renal biopsy results, a total of 20 patients were diagnosed with AAGN without LN. The remaining 39 patients were found to have LN + AAGN. Patients with LN + AAGN exhibited higher SLEDAI scores [14.00 (10.00–18.00) vs. 5.00 (2.50–12.50), *P* = 0.028]. In addition, the 24-h urinary protein level tended to be higher in the LN + AAGN group [3.10 (1.70, 6.69) g/d vs. 1.66 (0.88, 2.15) g/d, *p* = 0.066]. There was no statistically significant difference in BVAS between the two groups (Table 2).

**Table 2:** Comparison of clinical and laboratory characteristics between patients with LN + AAGN and those with AAGN alone.

Clinical characteristics	LN + AAGN (*N* = 39)	AAGN (*n* = 20)	*P*-value
Age, median (IQR)	45.00 (39.00, 71.00)	40.50 (30.25, 53.00)	0.197
Sex (Male/Female)	6/33	2/18	0.865
Race (Asian/Caucasian/African)	14/21/4	9/9/2	0.785
History of autoimmune disease (*n*, %)	6 (15.4%)	2 (10.0%)	0.865
Low C3	33 (94.3%)	13 (81.3%)	0.345
Low C4	20 (64.5%)	4 (40.0%)	0.318
MPO-ANCA/PR3-ANCA/double+	35/1/3	19/0/1	0.707
SLEDAI	14.00 (10.00, 18.00)	5.00 (2.50, 12.50)	0.028
BVAS	15.00 (12.00, 18.00)	15.00 (12.00, 17.00)	0.847
Laboratory tests			
WBC (×10^9^/L)	5.90 (4.60, 8.97)	5.20 (4.60, 12.90)	0.951
Platelets (×10^9^/L)	216.57 ± 64.60	172.67 ± 98.64	0.209
Hemoglobin (g/L)	80.08 ± 18.66	76.50 ± 24.49	0.599
CRP (mg/dL)	2.980 (0.50, 8.70)	1.27 (0.44, 8.38)	0.787
ESR (mm/h)	99.00 (70.50, 140.00)	57.00 (45.25, 75.50)	0.106
Creatinine (µmol/L)	285.71 (209.79, 572.39)	319.87 (218.59, 356.10)	0.858
eGFR[ml/(min × 1.73m^2^)]	17.35 (6.98, 28.50)	16.50 (13.00, 23.75)	0.827
Proteinuria (g/24 h)	3.10 (1.70, 6.69)	1.66 (0.88, 2.15)	0.066
ANA titer	640.00 (160.00, 1,280.00)	320.00 (200.00, 640.00)	0.28

LN + AAGN, overlapping lupus nephritis and antineutrophil cytoplasmic antibody-associated glomerulonephritis; AAGN, antineutrophil cytoplasmic antibody-associated glomerulonephritis; SD, standard deviation; SLEDAI, systemic lupus erythematosus disease activity index; BVAS, Birmingham vasculitis activity score; WBC, white blood cells; CRP, C-reactive protein; ESR, erythrocyte sedimentation rate; C3, complement 3; C4, complement 4; eGFR, estimated glomerular filtration rate; ANA, antinuclear antibody; ANCA, anti-neutrophil cytoplasmic antibodies; MPO, myeloperoxidase; PR3, proteinase 3.

### 3.3 Pathological characteristics between patients with LN + AAGN and those with AAGN alone

**Figure 2:**
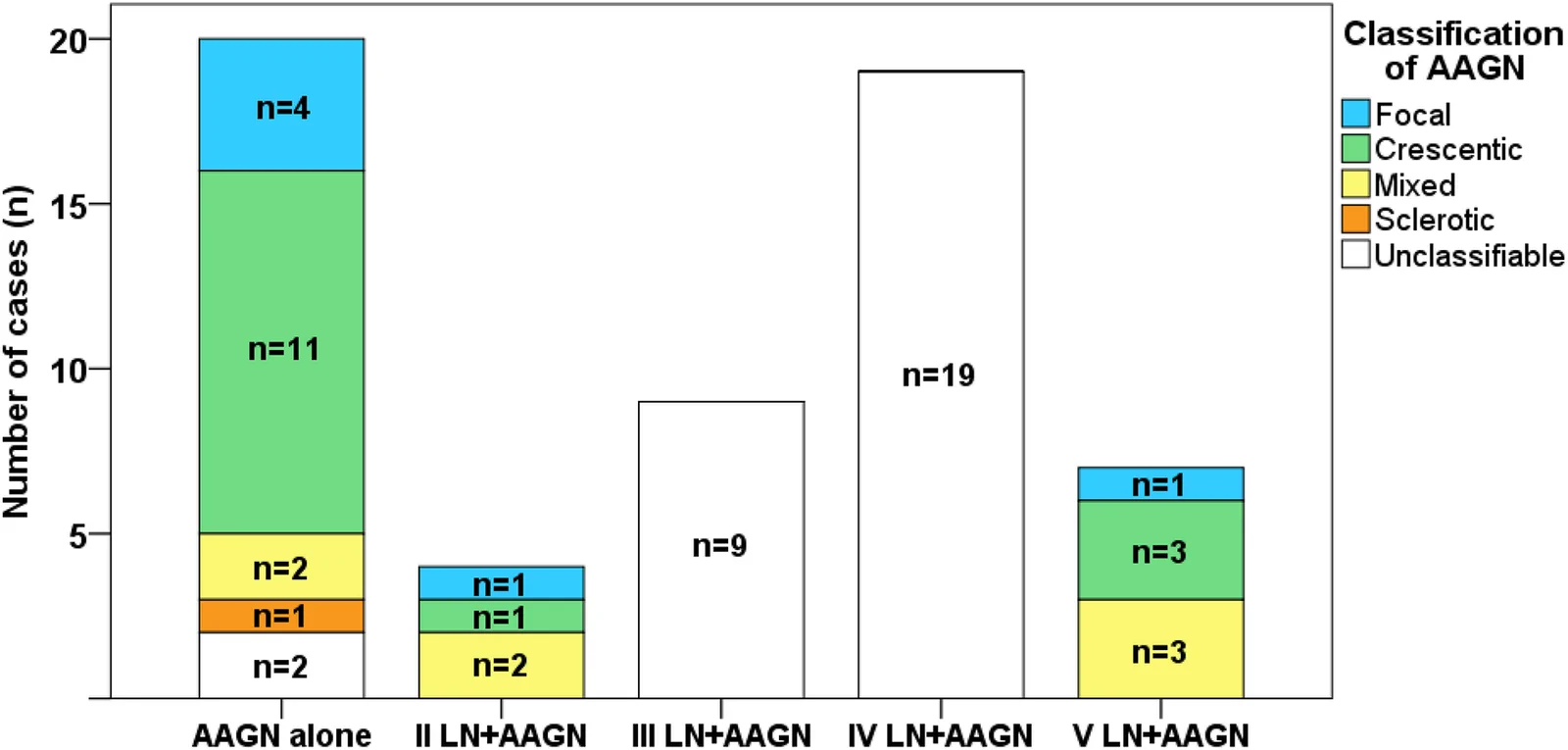
Distribution of renal biopsy pathological subtypes among 59 patients.

Among 20 patients diagnosed with AAGN without LN, 4 focal, 11 crescentic, 2 mixed, and 1 sclerotic were identified. Two patients could not be classified due to insufficient details in the original literature. When LN coexists with AAGN, the classification of AAGN becomes challenging. As types I, II, and V LN do not induce the formation of crescents, their coexistence with AAGN does not influence the classification of AAGN, whereas the coexistence of types III, IV, and VI LN makes the classification of AAGN impossible. Among 39 patients with LN + AAGN, 4 were diagnosed with LN II + AAGN (AAGN included one focal, one crescentic, and two mixed cases), 9 were diagnosed with LN III + AAGN, 19 with LN IV + AAGN, and 7 with LN V + AAGN (AAGN included one focal, three crescentic, and three mixed cases) (Figure 2).

### 3.4 Significance of elevated serum CRP levels

Elevated serum CRP levels are very common in active AAV, whereas serum CRP levels remain normal in most SLE patients. Thus, we analyzed the differences in the clinical manifestations between patients with elevated and normal serum CRP levels. Of the 59 patients, 24 had CRP data, with 15 showing elevated serum CRP levels and 9 showing normal serum CRP levels. The clinical and laboratory features of patients in the two groups are presented in Table 3. Patients with elevated CRP levels exhibited lower SLEDAI scores (9.11 ± 5.97 vs. 18.57 ± 8.60, *P* = 0.021) and higher blood leukocyte counts [(9.41 ± 3.75) × 10^9^/L vs. (4.24 ± 1.19) × 10^9^/L, *P* = 0.005]. There were no significant differences in other features between the two groups.

**Table 3:** Comparison of clinical and laboratory features between patients with elevated and normal serum CRP levels.

Clinical characteristics	Patients with elevated CRP (*n* = 15)	Patients with normal CRP (*n* = 9)	*P*-value
Age, mean ± SD	49.27 ± 19.55	43.33 ± 17.61	0.464
Sex (Male/Female)	3/12	0/9	0.426
Race (Asian/Caucasian/African)	8/7/0	6/2/1	0.255
History of autoimmune disease (*n*, %)	3 (20%)	4 (44.4%)	0.417
Low C3	14 (93.3%)	8 (88.9%)	1.000
Low C4	6 (46.2%)	5 (71.4%)	0.540
MPO-ANCA/PR3-ANCA/double+	13/0/2	9/0/0	0.253
SLEDAI	9.11 ± 5.97	18.57 ± 8.60	0.021
BVAS	15.00 (11.00, 15.00)	15.00 (13.50, 19.50)	0.142
Laboratory tests			
WBC (×10^9^/L)	9.41 ± 3.75	4.24 ± 1.19	0.005
Platelets (×10^9^/L)	215.80 ± 80.175	206.17 ± 84.051	0.822
Hemoglobin (g/L)	81.36 ± 23.45	87.78 ± 16.94	0.486
ESR (mm/h)	68.50 (50.75, 99.00)	106.00 (83.25, 140.00)	0.109
Creatinine (µmol/L)	285.00 (97.24, 328.71)	310.00 (129.60, 565.76)	0.194
eGFR [ml/(min × 1.73m^2^)]	17.00 (15.00, 61.50)	17.00 (7.00, 25.00)	0.238
Proteinuria (g/24 h)	1.65 ± 1.08	4.19 ± 3.15	0.086
ANA titer	320.00 (160.00, 2,560.00)	320.00 (115.00, 1,120.00)	0.382

CRP, C-reactive protein; SD, standard deviation; C3, complement 3; C4, complement 4; SLEDAI, systemic lupus erythematosus disease activity index; BVAS, Birmingham vasculitis activity score; WBC, white blood cells; ESR, erythrocyte sedimentation rate; eGFR, estimated glomerular filtration rate; ANA, antinuclear antibody; ANCA, anti-neutrophil cytoplasmic antibodies; MPO, myeloperoxidase; PR3, proteinase 3.

### 3.5 The relationship between peripheral leukocyte levels and clinical manifestations

Leucopenia is very common in SLE, whereas in AAV, the peripheral leukocyte counts usually increase. Therefore, we analyzed the differences in clinical and laboratory features among patients with high, normal, and low peripheral leukocyte counts. Of the 59 patients, 24 had peripheral leukocyte data and were divided into three groups (high, normal, and low). The 24-h urine protein levels in patients with low leukocyte counts (6.95 ± 3.61 g) were significantly higher than those in patients with normal counts (2.55 ± 1.78 g) and high counts (1.39 ± 0.98 g) (*P* = 0.029). The incidence of intra-alveolar hemorrhage was higher in patients with high leukocyte counts (80.00%) compared to those with normal (12.50%) or low counts (0.00%) (*P* = 0.016) (Table 4).

**Table 4:** Comparison among patients with different levels of leukocyte counts.

Clinical characteristics	Patients with high WBC (*n* = 5)	Patients with normal WBC (*n* = 16)	Patients with low WBC (*n* = 3)	*P*-value
Age, mean ± SD	52.20 ± 14.26	45.63 ± 23.99	43.00 ± 10.58	0.797
Sex (Male/Female)	0/5	2/14	0/3	0.580
Race (Asian/Caucasian/African)	3/2/0	11/4/1	3/0/0	0.708
History of autoimmune disease (*n*, %)	1 (20.00%)	3 (18.8%)	1 (33.33%)	0.849
Low C3	4 (80.00%)	14 (87.5%)	3 (100.00%)	0.710
Low C4	3 (60.00%)	6 (42.9%)	1 (33.33%)	0.725
Laboratory tests				
Platelets (×10^9^/L)	281.33 ± 73.39	202.53 ± 69.26	155.00 ± 36.35	0.088
Hemoglobin (g/L)	76.00 ± 22.34	82.00 ± 23.50	84.00 ± 13.53	0.848
Proteinuria (g/24 h)	1.39 ± 0.98	2.55 ± 1.78	6.95 ± 3.61	0.029
Clinical manifestations				
Intra-alveolar hemorrhage	4 (80.00%)	2 (12.5%)	0 (0.00%)	0.016

WBC, white blood cells; SD, standard deviation; C3, complement 3; C4, complement 4.

### 3.6 Correlation analysis of BVAS and SLEDAI with laboratory parameters in patients

**Figure 3:**
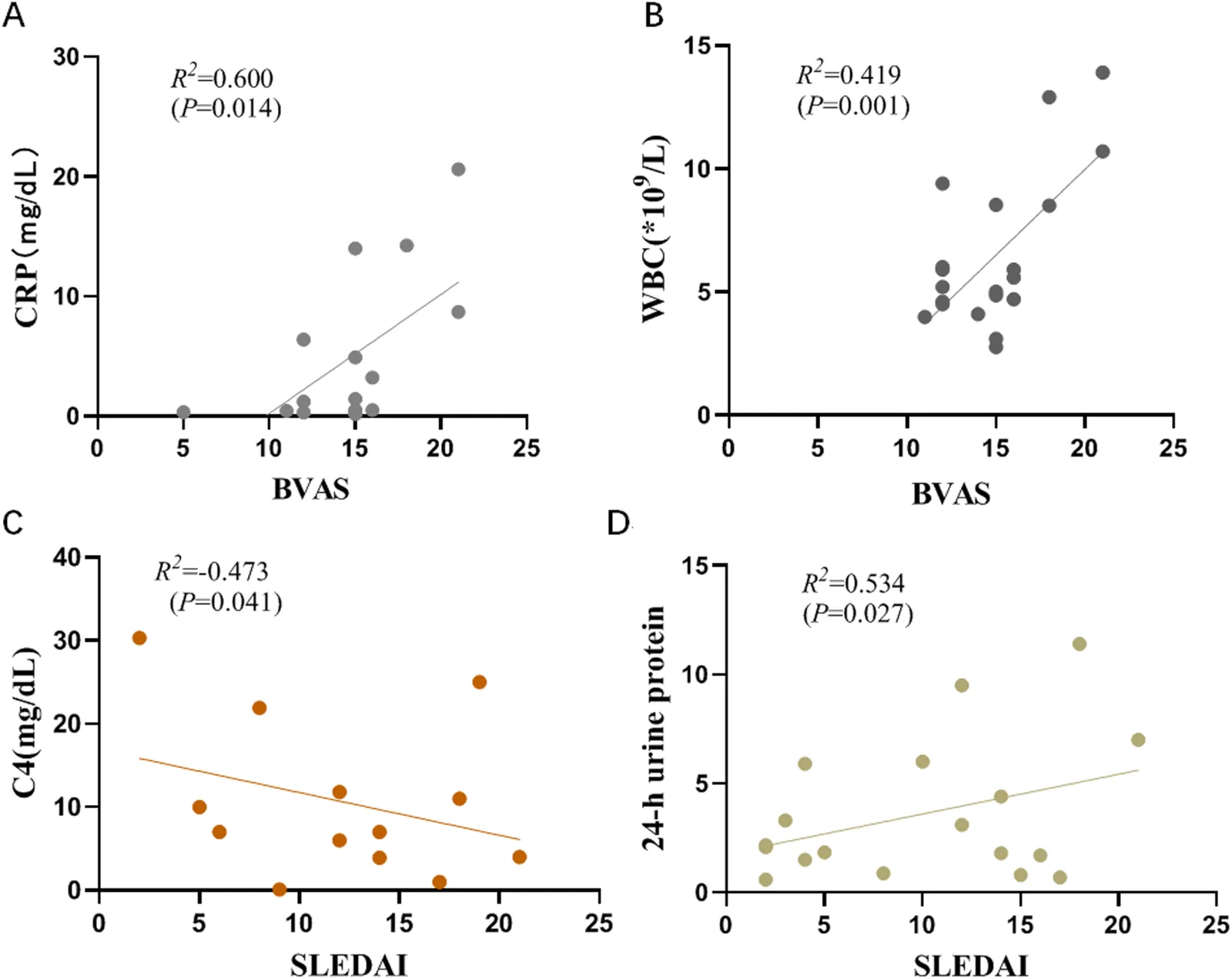
**(A)** Correlation between Birmingham vasculitis activity score (BVAS) and C-reactive protein (CRP). **(B)** BVAS and white blood cell (WBC). **(C)** Systemic lupus erythematosus disease activity index (SLEDAI) and 24-h urine protein. **(D)** SLEDAI and 24-h urine protein.

To find whether there was an association between the activity of SLE and AAV, we performed a correlation analysis of BVAS, SLEDAI scores, and other parameters. No significant correlation was found between BVAS and SLEDAI scores (*P* = 0.778). BVAS was positively correlated with CRP levels and blood leukocyte levels (*r* = 0.600, *P* = 0.014; *r* = 0.419, *P* = 0.001, respectively). SLEDAI scores were positively correlated with 24-h urine protein levels (*r* = 0.534, *P* = 0.027) and negatively correlated with C4 levels (*r* = − 0.473, *P* = 0.041) (Figure 3). Notably, no statistically significant association was detected between SLEDAI scores and complement C3 levels (*P* = 0.109).

### 3.7 Therapy and prognosis analysis

**Figure 4:**
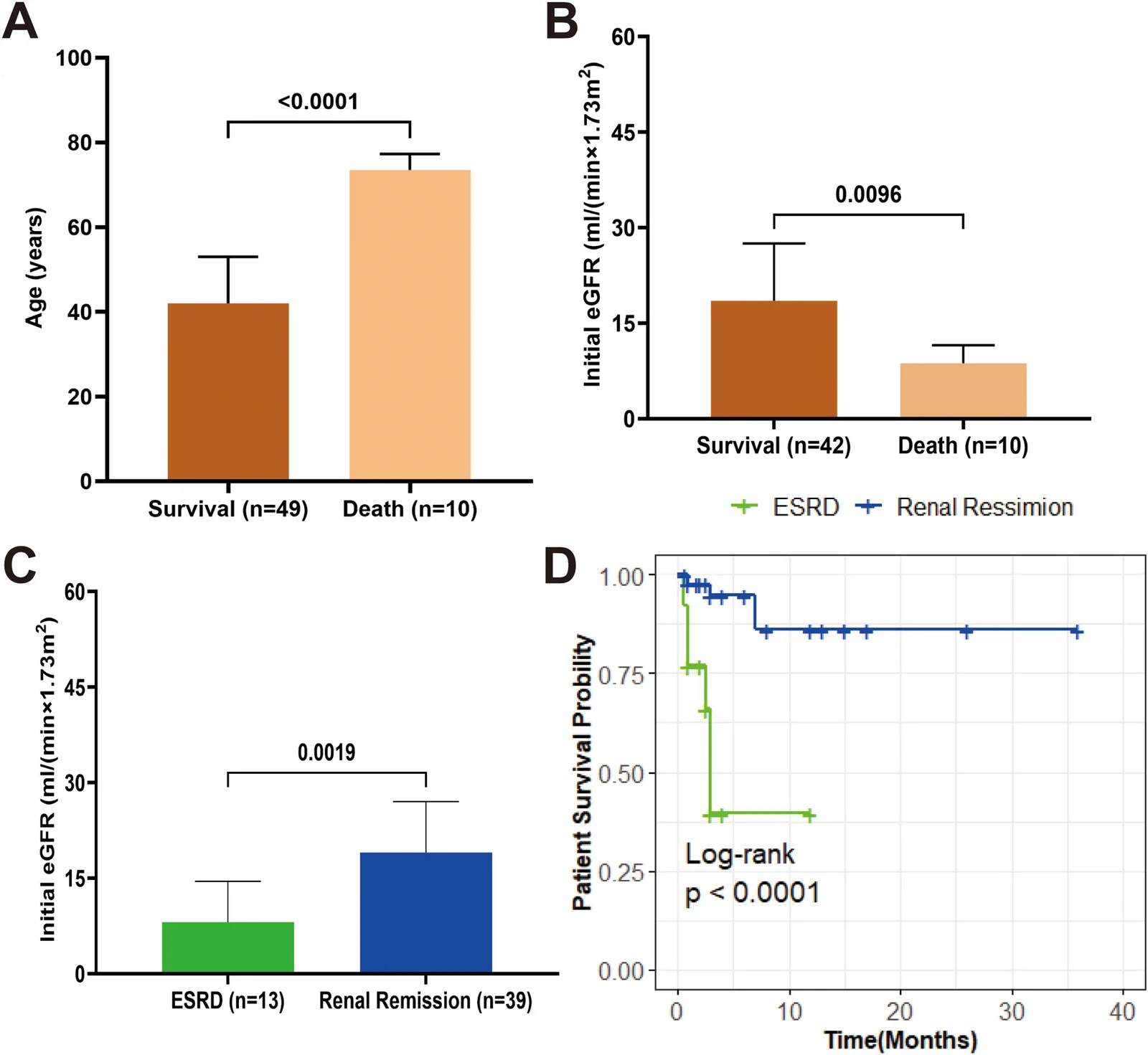
**(A,B)** Comparison of the mean age and the initial estimated glomerular filtration rate (eGFR) between the death group and survival group. **(C)** Compared with patients with preserved renal function, those who entered end-stage renal disease (ESRD) had lower initial eGFR levels. **(D)** Kaplan–Meier survival analysis indicated a statistically significant difference in survival time between the two groups.

The mean duration of follow-up was 3.0 months (ranging from 0.5 to 36 months). All patients received glucocorticoid + immunosuppressant therapy. Pulse corticosteroid therapy was administered to a total of 44 patients. Immunosuppressants included cyclophosphamide (*n* = 45), mycophenolate mofetil (*n* = 14), azathioprine (*n* = 8), rituximab (*n* = 7), hydroxychloroquine (*n* = 8), and tacrolimus (*n* = 4). Seventeen patients received plasma exchange. Of the 59 patients, 10 died during follow-up. The mean initial age of the patients who died [73.50 (55.00, 77.25) years] was significantly older than those who survived [42.00 (28.00, 53.00) years] (*P* < 0.001) (Figure 4A). Additionally, the initial eGFR levels of the patients who died during follow-up were significantly lower than those who survived [8.70 (5.75–11.5) vs. 18.50 (IQR, 9.25–27.5) mL/min/1.73 m^2^, *P* = 0.0096]. Patients who progressed to ESRD had lower initial eGFR levels [8.09 (5.00–14.50) vs. 19.00 (IQR, 11.00–27.00) mL/min/1.73 m^2^, *P* = 0.0019] (Figure 4C). The mortality rate in the ESRD group was 50.00%, while it was 6.70% in patients who did not progress to ESRD. Kaplan–Meier survival analysis revealed a statistically significant difference (*P* < 0.001) (Figure 4D).

Table 5 showed the comparison of therapy and prognosis between patients with LN + AAGN and those with AAGN alone. One patient lacked detailed treatment documentation. No statistically significant differences in therapeutic strategy were observed between the two groups. However, patients with LN + AAGN tended to receive more mycophenolate mofetil, rituximab, and plasma exchange, whereas cyclophosphamide was slightly more commonly used in patients with AAGN alone. The proportion of patients progressing to ESRD did not differ significantly between groups (23.1% vs. 25.0%, *p* = 1.000). The mortality rate was 23.1% in the LN + AAGN group and 5.0% in the AAGN alone group. The relative risk was 4.62 (95% CI: 0.63–33.91).

**Table 5:** Comparison of the therapy and outcomes between patients with LN + AAGN and those with AAGN alone.

Therapy/outcomes	LN + AAGN (*N* = 39*)	AAGN (*n* = 20)	*P*-value
Corticosteroid (Y/N)	37/1	20/0	1.000
Pulse corticosteroid therapy (Y/N)	24/14	11/9	0.546
Cyclophosphamide (Y/N)	28/10	17/3	0.515
Mycophenolate mofetil (Y/N)	11/27	3/17	0.391
Azathioprine (Y/N)	4/34	4/16	0.553
Tacrolimus (Y/N)	3/35	1/19	1.000
Rituximab (Y/N)	6/32	1/19	0.438
Hydroxychloroquine (Y/N)	6/32	2/18	0.836
Plasmapheresis (Y/N)	14/24	3/17	0.082
ESRD (Y/N)	9/30	5/15	1.000
Death (Y/N)	9/30	1/19	0.166

AAGN, antineutrophil cytoplasmic antibody-associated glomerulonephritis; ESRD, end stage renal disease.

* Treatment data were available for 38 out of 39 patients with LN + AAGN. LN + AAGN: Overlapping lupus nephritis and antineutrophil cytoplasmic antibody-associated glomerulonephritis.

## 4 Discussion

One important characteristic of SLE is the presence of various autoantibodies in serum. Most autoantibodies only indicate the severity of SLE, but some other autoantibodies suggest the possibility of concurrent other diseases. SLE can coexist with other autoimmune diseases such as antiphospholipid antibody syndrome, Sjögren's syndrome (SS), rheumatoid arthritis (RA), and AAV. SLE and AAV share similar clinical manifestations, such as fever, arthritis, skin lesions, and kidney injury. Renal biopsy is the best method for distinguishing LN from AAGN. LN is characterized by immune-complex deposition in the glomeruli, whereas AAGN typically shows an absence or slight immune-complex deposition with necrotizing crescentic glomerulonephritis (55, 56). In a study conducted by Wang Y et al., ANCA-positive SLE patients were older at diagnosis, demonstrated lower eGFR levels, and showed slower proteinuria remission following treatment compared with ANCA-negative SLE patients (2). Furthermore, ANCA-positive LN exhibited higher proportions of necrosis, crescent formation, and glomerulosclerosis, with lower immune complex deposition compared with ANCA-negative LN. These phenomena indicate that at least a part of ANCA-positive SLE patients exhibit characteristics resembling AAGN. However, this study did not distinguish AAGN from LN. To the best of our knowledge, our research is the first systematic study focusing on the features of SLE combined with renal biopsy-proven AAGN.

ANCA recognizes more than 10 types of target antigens. When studying the significance of ANCA in SLE, the first step is to determine the type of antigen recognized by ANCA. Unlike primary AAV, the antigen recognition spectrum of ANCA in SLE is more heterogeneous. Manolova I et al. reported that 29.10% (16/55) SLE patients had positive ANCA, with MPO-ANCA found in 10.90% (6/55), lactoferrin (LF)-ANCA in 18.20% (10/55), PR3-ANCA in 12.70% (7/55), bactericidal/permeability-increasing protein (BPI)-ANCA in 23.60% (13/55), and elastase (HLE)-ANCA in 1.80% (1/55) (57). The characteristics of primary AAV differ between Asians and Caucasians. Among Caucasian patients with primary AAV, the positivity rates of MPO-ANCA and PR3-ANCA are similar. Although Caucasian patients with MPO-ANCA exhibit more severe kidney injury than those with PR3-ANCA, the difference is not as obvious as in Asians (58). In the current study, MPO-ANCA-positive patients account for the vast majority, but Asians constitute only 38.98% of the cohort. Thus, in both Asians and Caucasians, MPO-ANCA is overwhelmingly predominant in SLE patients with renal biopsy-proven AAGN.

Due to the complex pathological manifestations of LN, diagnosing AAGN in the background of LN is a challenging task. When LN is severe and AAGN is slight (e.g., only presenting with some fibrinoid necrosis), AAGN can easily be covered by the co-existing LN. Thus, specific criteria should be followed to identify the coexistence of LN and AAGN. The first case reported in our study presented with clinical features of SLE, weak “full-house” glomerular deposits, and slight focal proliferative lesions, but prominent features of fibrinoid necrosis and crescent formation, suggesting that the renal involvement was primarily caused by AAGN with concurrent mild LN (type III). The second case also met the clinical diagnostic criteria for SLE but exhibited only sparse immune complex deposition in the renal biopsy, leading to a diagnosis of AAGN without LN. By combining our two cases with 57 additional cases enrolled from the literature, we found that up to 33.90% of SLE patients with renal biopsy-proven AAGN did not have concurrent LN. We found that SLEDAI scores was significantly higher in patients with LN + AAGN compared to those with AAGN alone, and the 24-h urinary protein level tended to be higher in the LN + AAGN group. However, the initial eGFR and renal prognosis did not differ between the two groups. Thus, for SLE patients with positive ANCA, especially MPO-ANCA, if the proteinuria is relatively slight but renal insufficiency is disproportionately severe, the existence of AAGN should be considered.

Elevated CRP levels and peripheral WBC counts are two important features of primary AAV. In the current study, no differences in CRP levels were observed between patients with LN + AAGN and those with AAGN alone, but CRP levels were positively correlated with peripheral WBC counts. Therefore, patients with elevated CRP levels have more clinical characteristics of AAV. We also found that patients with elevated peripheral WBC counts had lower urinary protein levels and a significantly higher incidence of pulmonary hemorrhage compared to patients with decreased WBC counts.

In this study, we calculated SLEDAI and BVAS for each patient. It is important to note that calculating SLEDAI and BVAS for patients with SLE + AAV is not easy due to the overlapping clinical manifestations of SLE and AAV. We adhered to the following principles: If a clinical manifestation can be explained by either SLE or AAV, it is included in both scoring systems. For patients with LN in renal biopsies, kidney injury scores were integrated into both SLEDAI and BVAS. For patients with no evidence of LN in renal biopsies, kidney injury scores were integrated only into BVAS. We acknowledge that this principle is not entirely reasonable, but it still helps answer some of the questions we sought to address. The results of statistical analysis revealed that SLEDAI scores were correlated with proteinuria and C4 levels, while BVAS was correlated with CRP and WBC levels. Since mutual interference cannot be avoided when calculating SLEDAI and BVAS separately, the significance of these results should be interpreted with caution.

Currently, there is no standardized treatment protocol for patients with overlapping SLE and AAV. Most patients underwent induction therapy consisting of steroids and cyclophosphamide. Among the 59 patients evaluated, 14 progressed to ESRD, and 10 died, indicating a relatively poor prognosis for this cohort. Patients who died were older and had lower initial eGFR levels at disease onset. Survival analysis revealed that patients who progressed to ESRD had a higher mortality rate than those with preserved renal function. The primary cause of death in most cases was severe infection. Therefore, for patients with severe renal insufficiency, it is crucial to appropriately adjust the intensity of immunosuppressive therapy to minimize the risk of lethal infection.

According to the latest international guideline recommendations, the treatment principles for LN and AAGN are not the same (59–62). However, when comparing the therapeutic regimens received by patients with LN + AAGN and those with AAGN alone, we found no statistically significant difference between the two groups. Given that the patients enrolled in this study were derived from different countries and spanned a long time period, many of them appeared not to have received sufficiently standardized treatment—for example, hydroxychloroquine administration was remarkably infrequent in our cohort, even though hydroxychloroquine is a well-established foundational therapy for SLE. Furthermore, according to the latest guidelines, biological agents are playing an increasingly important role in the management of LN and AAGN; nevertheless, in our study, except for a few patients who received rituximab, no newer biological agents were used. For ANCA-positive SLE patients, renal injury could be attributed to LN, pauci-immune AAGN, or overlapping lesions of the two, which may bring different implications for treatment. Treatments could tend toward LN protocols when LN lesions predominate, while AAV-related treatment schemes could be considered for cases dominated by pauci-immune necrotizing/crescentic glomerulonephritis. Cases with overlapping pathology may require customized treatment plans. Renal biopsy helps judge the main pathological changes and facilitates therapeutic choices.

Some limitations of this study should be mentioned. First, the total sample size and number of endpoint events were small, which reduced statistical power. Second, this study focused specifically on the impact of AAGN on SLE, so we included only patients with biopsy-proven renal AAGN, excluding those diagnosed by classification criteria alone or by other biopsies (e.g., lung) (63). Hence, our results may not reflect all SLE-AAV overlap cases. Third, SLEDAI and BVAS of some patients were assessed retrospectively without prospective unified evaluation, which brought unavoidable bias; we could not accomplish detailed pathological subtype classification of AAGN for most patients with concurrent LN and AAGN due to insufficient renal pathological descriptions in published studies. Forth, treatment regimens varied greatly among different groups, countries and time periods, potentially affecting outcome interpretation. Moreover, due to disease rarity, most patients came from published reports with incomplete data, so further validation is needed in future.

## 5 Conclusion

For ANCA-positive SLE patients, renal impairment may be caused by LN, LN combined with AAGN, or even isolated AAGN. Renal biopsy is crucial as it helps clinicians select appropriate treatment regimens.

## Data Availability

The datasets presented in this study can be found in online repositories. The names of the repository/repositories and accession number(s) can be found in the article/Supplementary Material.
