# Supplementary material for: Clinical features of concurrent systemic lupus erythematosus and renal biopsy-proven antineutrophil cytoplasmic antibody-associated vasculitis: a retrospective analysis
Source: Front Med (Lausanne). 2026 Sep 9;13:1842903. doi: 10.3389/fmed.2026.1842903 (PMC13597386; doi:10.3389/fmed.2026.1842903)
Supplement: Supplementary file 2 [file Table3.docx]

| Supplementary Table 3. Search Strategies Used for Each Database | |  |  |
| --- | --- | --- | --- |
| Database | Search Terms | Date Range | Number of publications |
| PubMed | (((Systemic Lupus Erythematosus OR Lupus) AND (Anti-Neutrophil Cytoplasmic Antibody-Associated Vasculitis OR ANCA-associated vasculitis OR Antineutrophil Cytoplasmic Antibody OR ANCA OR granulomatosis with polyangiitis OR Wegener's granulomatosis OR microscopic polyangiitis OR eosinophilic granulomatosis with polyangiitis OR Churg-Strauss syndrome OR pauci-immune glomerulonephritis OR crescentic glomerulonephritis)) OR (SLE/AAV overlap syndrome)) AND (("1990/01/01"[Date - Publication] : "2026/07/01"[Date - Publication])) | Between January 1, 1990, and July 1, 2026 | 2336 |
| Web of Science | "Systemic Lupus Erythematosus" OR "Lupus" (Topic) and "Anti-Neutrophil Cytoplasmic Antibody-Associated Vasculitis" OR "ANCA-associated vasculitis" OR "Antineutrophil Cytoplasmic Antibody" OR "ANCA" OR "granulomatosis with polyangiitis" OR "Wegener's granulomatosis" OR "microscopic polyangiitis" OR "eosinophilic granulomatosis with polyangiitis" OR "Churg-Strauss syndrome" OR "pauci-immune glomerulonephritis" OR "crescentic glomerulonephritis" (Topic) or "SLE/AAV overlap syndrome" (Topic) | Between January 1, 1990, and July 1, 2026 | 3720 |
| Embase | (('systemic lupus erythematosus':ti,ab,kw OR 'lupus':ti,ab,kw) AND ('antineutrophil cytoplasmic antibody':ti,ab,kw OR 'anti-neutrophil cytoplasmic antibody-associated vasculitis':ti,ab,kw OR 'anca':ti,ab,kw OR 'wegener granulomatosis':ti,ab,kw OR 'microscopic polyangiitis':ti,ab,kw OR 'eosinophilic granulomatosis with polyangiitis':ti,ab,kw OR 'churg-strauss syndrome':ti,ab,kw OR 'pauci-immune glomerulonephritis':ti,ab,kw OR 'crescentic glomerulonephritis':ti,ab,kw) OR 'sle/aav overlap syndrome':ti,ab,kw) AND [1990-2026]/py AND [01-01-1990]/sd NOT [02-07-2026]/sd | Between January 1, 1990, and July 1, 2026 | 3235 |
| China National Knowledge Infrastructure | （主题：(系统性红斑狼疮+狼疮性肾炎)）AND（主题：(抗中性粒细胞胞质抗体相关性血管炎+ANCA+寡免疫)）OR （主题：(SLE/AAV重叠综合征)） | Between January 1990 and July 2026 | 287 |
| Wanfang Data | 检索表达式（中英文扩展）： (主题:(系统性红斑狼疮) and 主题:(ANCA相关血管炎) or 主题:(SLE\/AAV重叠综合征)) and 发表时间:1990-2026 | Between January 1990 and July 2026 | 142 |
